# Supplementary material for: Affect and post-COVID-19 symptoms in daily life: An exploratory experience sampling study
Source: PLoS One. 2024 Oct 24;19(10):e0295217. doi: 10.1371/journal.pone.0295217 (PMC11500883; doi:10.1371/journal.pone.0295217)
Supplement: S1 File — (PDF) [file pone.0295217.s001.pdf]

Table S1. *Morning Survey: Provided Once a Day; Available Between 08.00 And 12.00*

Question	Type of question	Response options
**Kunt u op onderstaande lijn aangeven hoe uw gezondheid gisteren was? *(Can you indicate on the below line yesterdays' health status?)***	VAS	0: slechte gezondheid – 10: goede gezondheid *(0: bad health – 10: good health)*
**Hoeveel uur heeft u vannacht geslapen? *(How many hours of sleep did you get last night?)***	SAQ	1: Minder dan 4 uur *(less than 4 hours)*<br>2: Tussen 4 en 6 uur *(between 4 and 6 hours)*<br>3: Tussen 6 en 8 uur *(between 6 and 8 hours)*<br>4:Tussen 8 en 10 uur *(between 8 and 10 hours)*<br>5: Meer dan 10 uur *(over 10 hours)*
**In welke mate had u vannacht last van problemen met slapen? *(To what extent did you experience sleep difficulties last night?)***	SAQ	1: Geen last gehad *(No problems)*<br>2: Een beetje last gehad *(a little bit of trouble)*<br>3: Redelijk last gehad *(quite a bit of trouble)*<br>4: Behoorlijk last gehad *(a lot of trouble)*<br>5: Heel veel last gehad *(extreme trouble)*
**Heeft u gister overdag dutjes gedaan? *(Did you take any naps yesterday?)***	SAQ	Ja / Nee (Yes / No)<br>'Yes' opens up the next question, 'no' closes the survey
**Hoeveel uur heeft u gister overdag geslapen? (*How many hours did you nap yesterday?*)**	SAQ	1: Minder dan een half uur *(less than half an hour)*<br>2: Tussen een half uur en 1 uur *(between half an hour and 1 hour)*<br>3: Tussen 1 en 2 uur *(between 1 and 2 hours)*<br>4:Tussen 2 en 3 uur *(between 2 and 3 hours)*<br>5:Tussen 3 en 4 uur *(between 3 and 4 hours)*

		6:Tussen 4 en 6 uur *(between 4 and 6 hours)*<br>7: Meer dan 6 uur *(over 6 hours)*

Notes. SAQ = Single Answer Questionnaire; VAS = Visual Analogue Scale

Table S2. *State Survey: Provided Six Times a Day And Prompted at Semi-Random Times Between 08.00 And 20.00*

Question	Type of question	Response options
**Op dit moment voel ik mij lichamelijk vermoeid *(Right now, I feel physically fatigued)***	SAQ	1: Sterk mee oneens *(strongly disagree)* – 7: sterk mee eens *(strongly agree)*
**Op dit moment voel ik mij mentaal vermoeid (bijvoorbeeld een gebrek aan energie om goed over iets na te denken, plannen te maken of creatief te zijn) *(Right now, I feel mentally fatigued (for example a lack of energy to think thoroughly, make plans or be creative))***	SAQ	1: Sterk mee oneens *(strongly disagree)* – 7: sterk mee eens *(strongly agree)*
**Op dit moment voel ik mij kortademig *(Right now, I experience shortness of breath)***	SAQ	1: Sterk mee oneens *(strongly disagree)* – 7: sterk mee eens *(strongly agree)*
**Ervaart u op dit moment pijn in uw lichaam? *(Right now, do you experience any pain in your body?)***	SAQ	Ja / Nee *(Yes / No)*<br>'Yes' opens up the next four question, 'no' advances directly to the 'thinking' question
**In hoeverre heeft u op dit moment hoofdpijn? *(To what extent do you experience a headache right now?)***	VAS	0: Geen hoofdpijn *(no headache)* – 10: meest erge hoofdpijn *(worst imaginable headache)*
**In hoeverre heeft u op dit moment gewrichtspijn? *(To what extent do you experience joint pain right now?)***	VAS	0: Geen gewrichtspijn *(no joint pain)* – 10: meest erge gewrichtspijn *(worst imaginable joint pain)*

**In hoeverre heeft u op dit moment pijn/ongemak op de borst? *(To what extent do you experience discomfort or pain of the chest right now?)***	VAS	0: Geen pijn op de borst *(no chest pain)* – 10: meest erge pijn op de borst *(worst imaginable chest pain)*
**In hoeverre heeft u op dit moment pijn ergens anders in uw lichaam dan aan het hoofd, aan de gewrichten en/of op de borst? *(To what extent do you experience bodily pain elsewhere than in the head, in the joints and/or of the chest right now?)***	VAS	0: Geen pijn *(no pain)* – 10: meest erge pijn *(worst imaginable pain)*
**Hoe gaat het met uw 'denken' (concentratie, geheugen, aandacht) op dit moment? *(How is your 'thinking' (concentration, memory, attention) right now?)***	VAS	0: Moeilijk en langzaam *(difficult and slow)* – 10: scherp en alert *(sharp and alert)*
**Op dit moment voel ik mij angstig *(right now, I feel anxious)***	SAQ	1: Sterk mee oneens *(strongly disagree)* – 7: sterk mee eens *(strongly agree)*
**Op dit moment voel ik mij somber *(right now, I feel gloomy)***	SAQ	1: Sterk mee oneens *(strongly disagree)* – 7: sterk mee eens *(strongly agree)*
**Op dit moment voel ik mij verdrietig *(right now, I feel sad)***	SAQ	1: Sterk mee oneens *(strongly disagree)* – 7: sterk mee eens *(strongly agree)*
**Op dit moment voel ik mij prikkelbaar *(right now, I feel irritable)***	SAQ	1: Sterk mee oneens *(strongly disagree)* – 7: sterk mee eens *(strongly agree)*
**Op dit moment voel ik mij teleurgesteld *(right now, I feel disappointed)***	SAQ	1: Sterk mee oneens *(strongly disagree)* – 7: sterk mee eens *(strongly agree)*
**Op dit moment voel ik mij opgewekt *(right now, I feel excited)***	SAQ	1: Sterk mee oneens *(strongly disagree)* – 7: sterk mee eens *(strongly agree)*
**Op dit moment voel ik mij ontspannen *(right now, I feel relaxed)***	SAQ	1: Sterk mee oneens *(strongly disagree)* – 7: sterk mee eens *(strongly agree)*
**Op dit moment voel ik mij tevreden *(right now, I feel satisfied)***	SAQ	1: Sterk mee oneens *(strongly disagree)* – 7: sterk mee eens *(strongly agree)*

**Op dit moment voel ik mij dankbaar *(right now, I feel thankful)***	SAQ	1: Sterk mee oneens *(strongly disagree)* – 7: sterk mee eens *(strongly agree)*
**Op dit moment voel ik mij blij *(right now, I feel joyful)***	SAQ	1: Sterk mee oneens *(strongly disagree)* – 7: sterk mee eens *(strongly agree)*
**Vlak voor het invullen van de vragen was ik bezig met… *(Right before answering the questions, I was doing…)***	SAQ	1: Niets *(nothing)*<br>2: Inspannend ontspannen (bijvoorbeeld wandelen, fietsen, tuinieren) *(strenuous relaxation (for example walking, riding a bike, gardening))*<br>3: Passief ontspannen (bijvoorbeeld televisie kijken, boek lezen) *(passive relaxation (for example watching television, reading a book))*<br>4: Slapen of rusten *(sleeping or resting)*<br>5: Werk/school *(Work/study)*<br>6: Het huishouden *(household choires)*<br>7: Eten/drinken *(eating/drinking)*<br>8: zelfzorg (bijvoorbeeld lichaamshygiëne, medicatie) *(self-care (for example bodily hygiene, medication))*<br>9: Onderweg *(On the go)*<br>10: Sociaal contact *(social interaction)*<br>11: iets anders, namelijk… *(Something else, namely…)* – this option opens up the next question, all others close the survey
**Met welke activiteit was u vlak voor het invullen van de vragen bezig? *(What were you doing before answering the questions?)***	Open question	Not applicable

Notes. SAQ = Single Answer Questionnaire; VAS = Visual Analogue Scale
