# Supplementary material for: Affect and post-COVID-19 symptoms in daily life: An exploratory experience sampling study
Source: PLoS One. 2024 Oct 24;19(10):e0295217. doi: 10.1371/journal.pone.0295217 (PMC11500883; doi:10.1371/journal.pone.0295217)
Supplement: S2 File — (PDF) [file pone.0295217.s002.pdf]

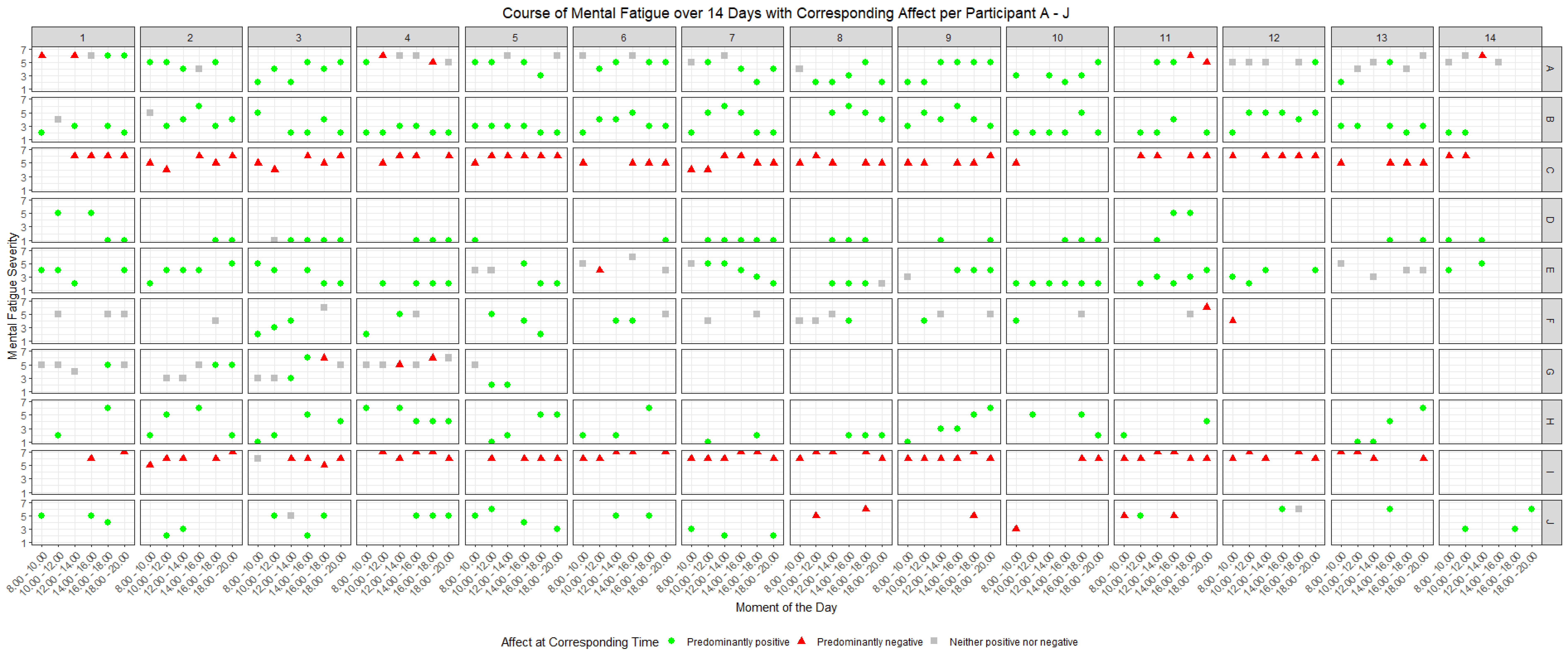

Course of Mental Fatigue over 14 Days with Corresponding Affect per Participant A - J
1
2
3
4
5
6
7
8
9
10
11
12
13
14
A
B
C
D
E
F
G
H
I
J
Mental Fatigue Severity
Moment of the Day
8.00 - 10.00
10.00 - 12.00
12.00 - 14.00
14.00 - 16.00
16.00 - 18.00
18.00 - 20.00
Affect at Corresponding Time
Predominantly positive
Predominantly negative
Neither positive nor negative

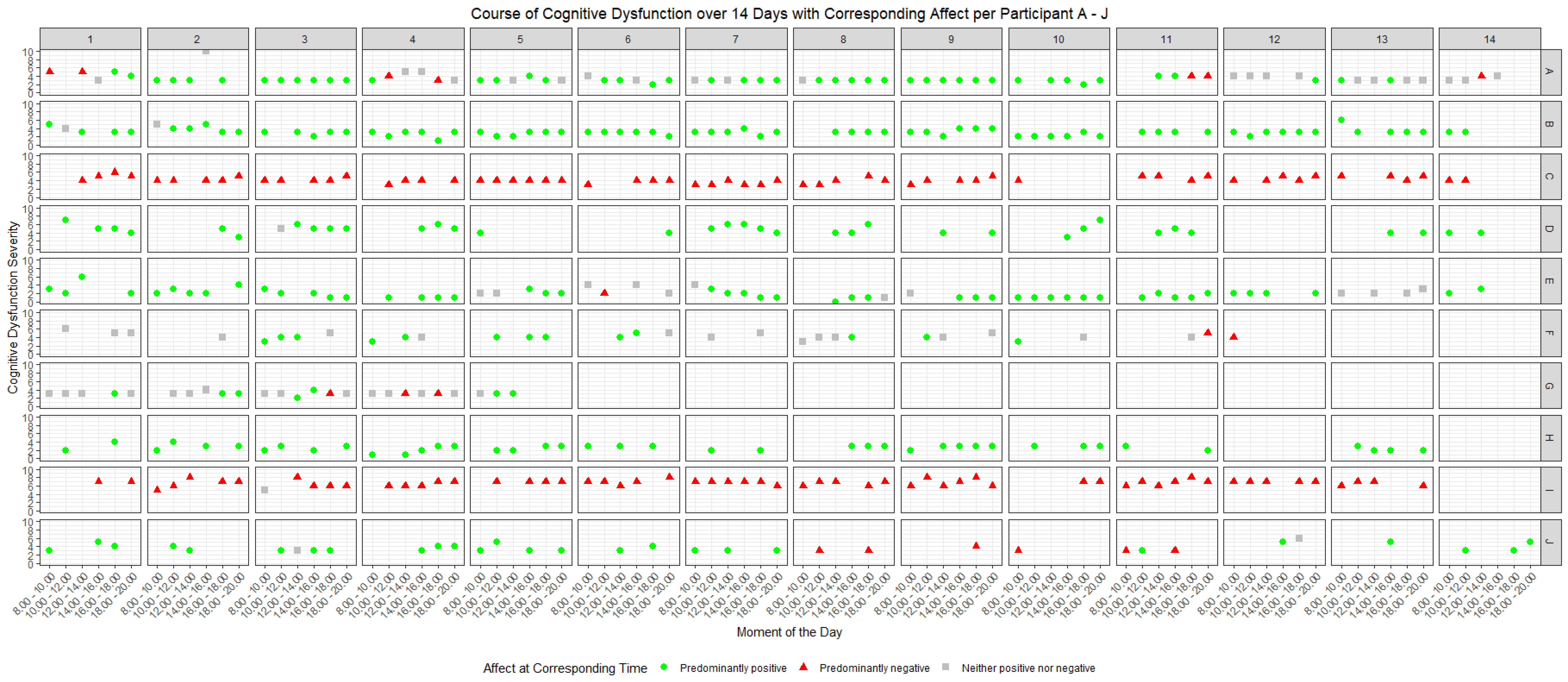

Course of Cognitive Dysfunction over 14 Days with Corresponding Affect per Participant A - J
1
2
3
4
5
6
7
8
9
10
11
12
13
14
A
B
C
D
E
F
G
H
I
J
Cognitive Dysfunction Severity
0
2
4
6
8
10
8.00 - 10.00
10.00 - 12.00
12.00 - 14.00
14.00 - 16.00
16.00 - 18.00
18.00 - 20.00
Moment of the Day
Affect at Corresponding Time
Predominantly positive
Predominantly negative
Neither positive nor negative

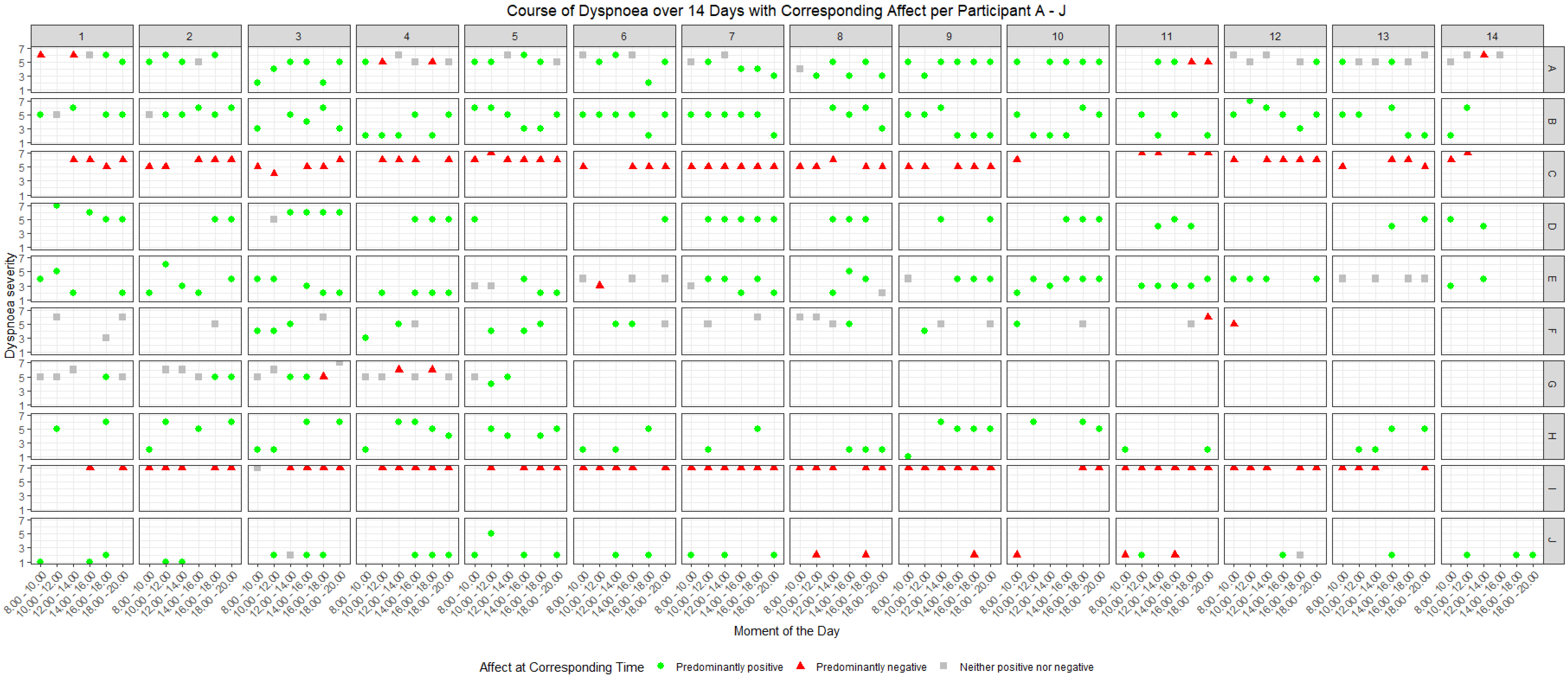
Course of Dyspnoea over 14 Days with Corresponding Affect per Participant A - J
1
2
3
4
5
6
7
8
9
10
11
12
13
14
A
B
C
D
E
F
G
H
I
J
Dyspnoea severity
1
3
5
7
8.00 - 10.00
10.00 - 12.00
12.00 - 14.00
14.00 - 16.00
16.00 - 18.00
18.00 - 20.00
Moment of the Day
Affect at Corresponding Time
Predominantly positive
Predominantly negative
Neither positive nor negative

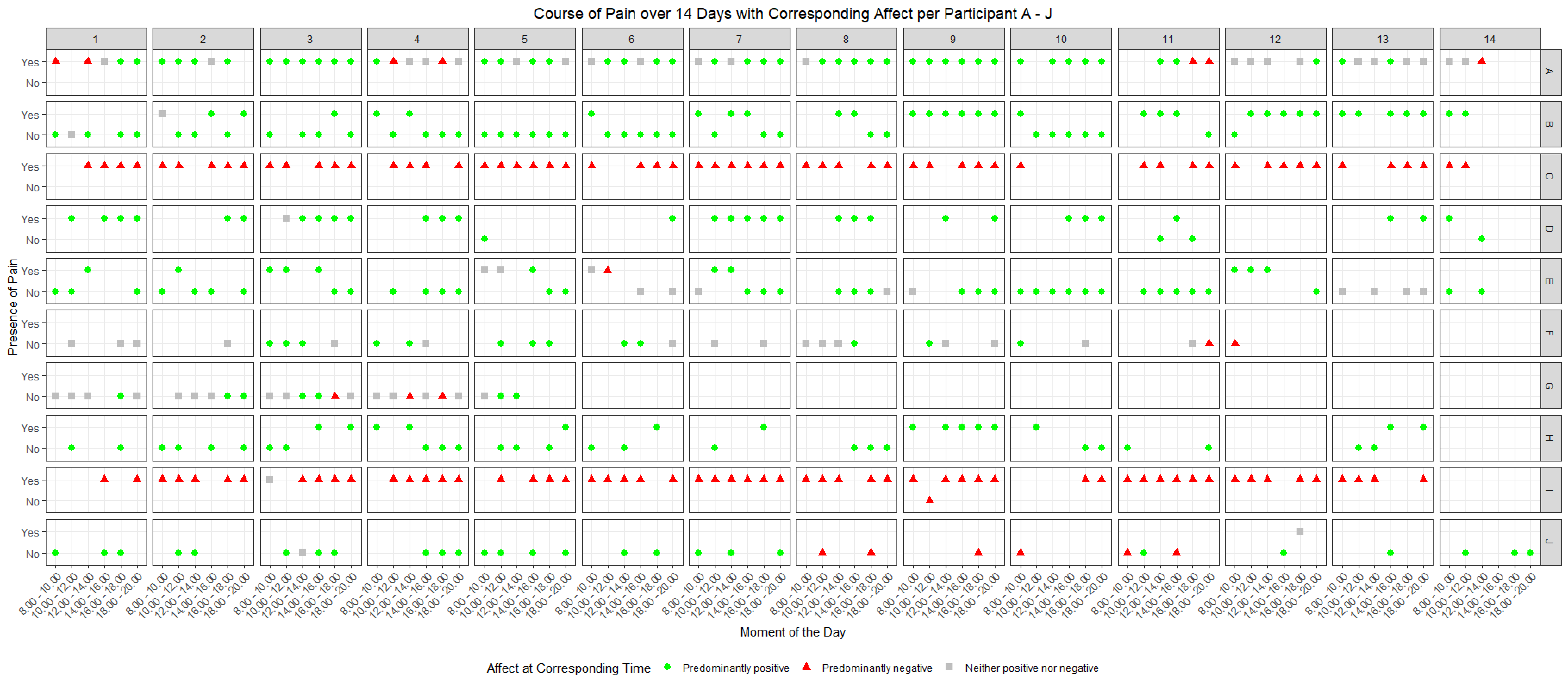

Course of Pain over 14 Days with Corresponding Affect per Participant A - J
1
2
3
4
5
6
7
8
9
10
11
12
13
14
A
B
C
D
E
F
G
H
I
J
Yes
No
Presence of Pain
8.00 - 10.00
10.00 - 12.00
12.00 - 14.00
14.00 - 16.00
16.00 - 18.00
18.00 - 20.00
Moment of the Day
Affect at Corresponding Time
Predominantly positive
Predominantly negative
Neither positive nor negative
