# Supplementary material for: Affect and post-COVID-19 symptoms in daily life: An exploratory experience sampling study
Source: PLoS One. 2024 Oct 24;19(10):e0295217. doi: 10.1371/journal.pone.0295217 (PMC11500883; doi:10.1371/journal.pone.0295217)
Supplement: S4 File — (PDF) [file pone.0295217.s004.pdf]

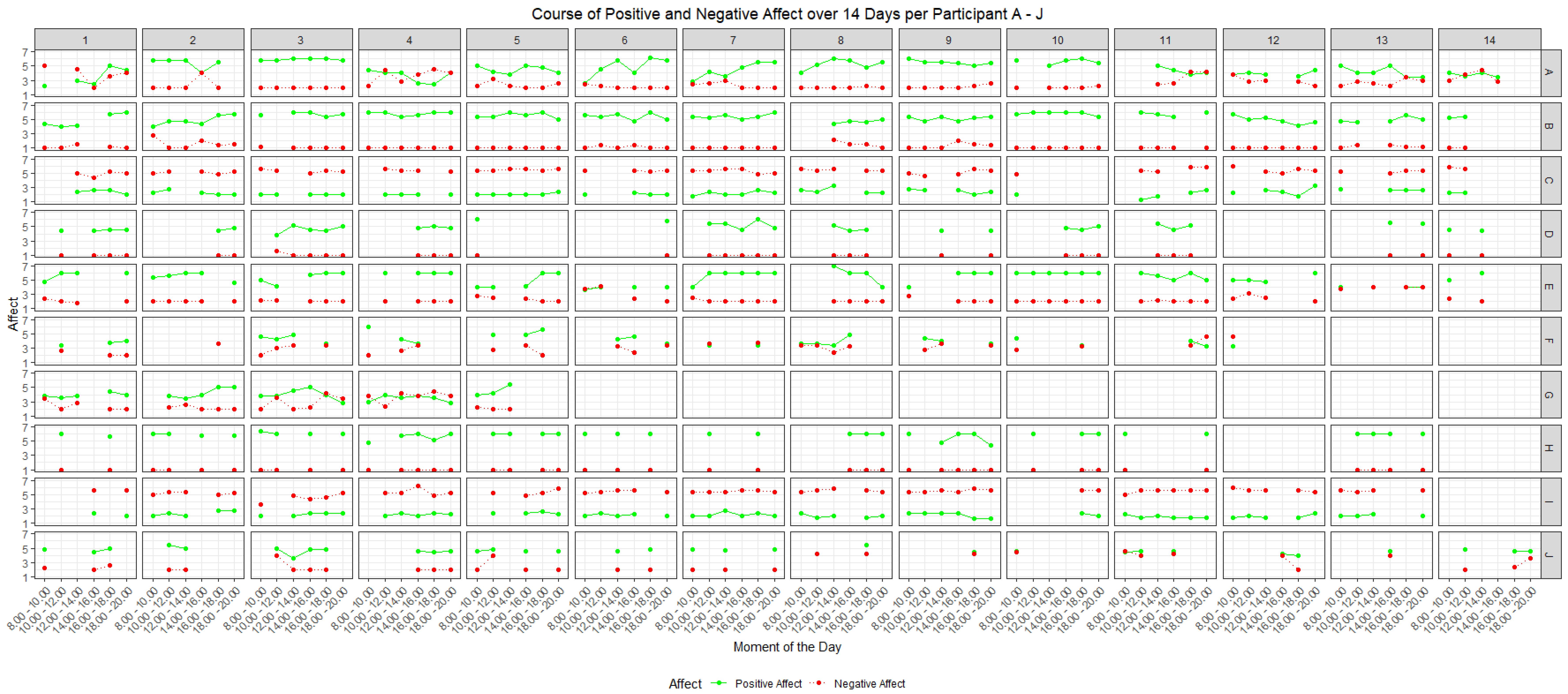

Course of Positive and Negative Affect over 14 Days per Participant A - J
1
2
3
4
5
6
7
8
9
10
11
12
13
14
A
B
C
D
E
F
G
H
I
J
Affect
Moment of the Day
8.00 - 10.00
10.00 - 12.00
12.00 - 14.00
14.00 - 16.00
16.00 - 18.00
18.00 - 20.00
Affect
Positive Affect
Negative Affect

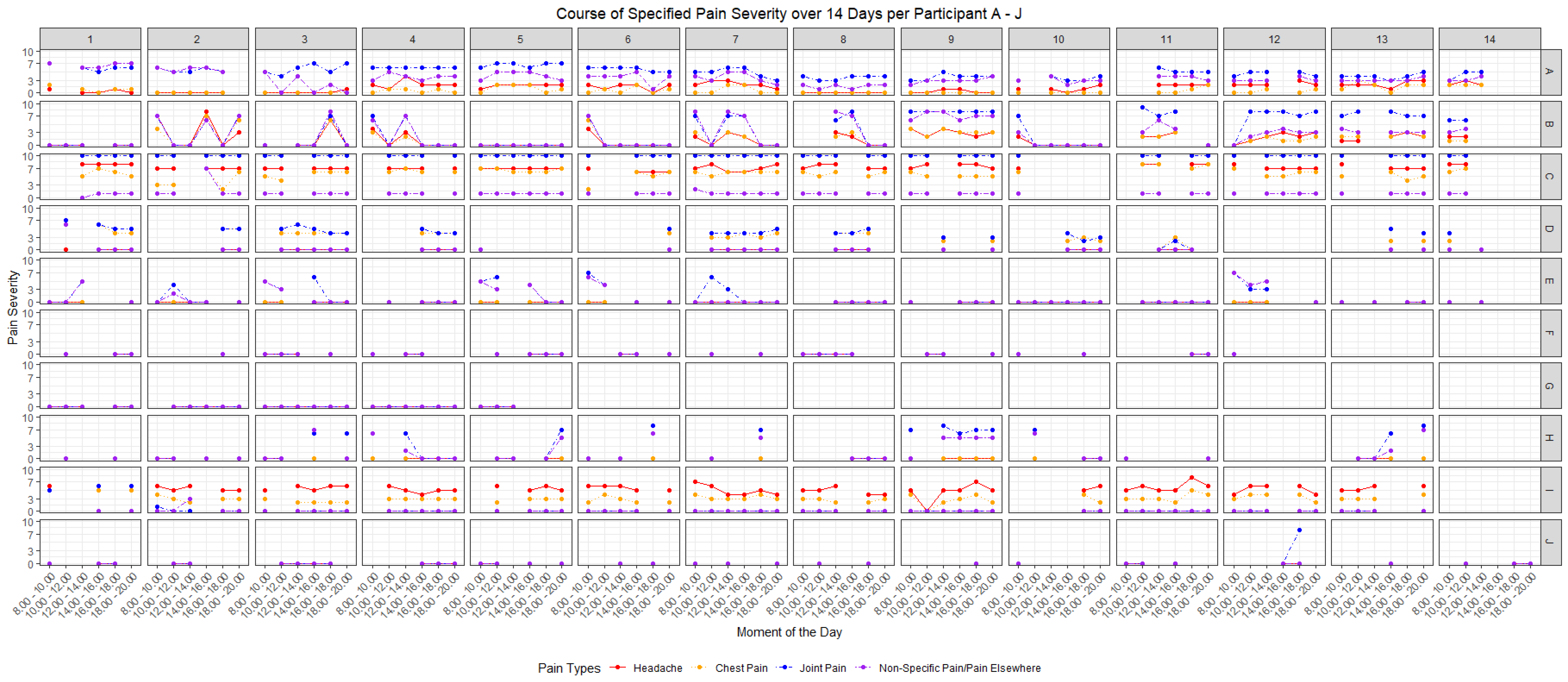

Course of Specified Pain Severity over 14 Days per Participant A - J
1
2
3
4
5
6
7
8
9
10
11
12
13
14
A
B
C
D
E
F
G
H
I
J
Pain Severity
10
7
3
0
8.00 - 10.00
10.00 - 12.00
12.00 - 14.00
14.00 - 16.00
16.00 - 18.00
18.00 - 20.00
Moment of the Day
Pain Types
Headache
Chest Pain
Joint Pain
Non-Specific Pain/Pain Elsewhere
