# Supplementary material for: Affect and post-COVID-19 symptoms in daily life: An exploratory experience sampling study
Source: PLoS One. 2024 Oct 24;19(10):e0295217. doi: 10.1371/journal.pone.0295217 (PMC11500883; doi:10.1371/journal.pone.0295217)
Supplement: Attachment — Submitted filename: Responses to Reviewers.docx [file pone.0295217.s005.docx]

Thank you for your time and for inviting us to revise our manuscript, *Affect and post-COVID-19 symptoms in daily life: An exploratory experience sampling study*. We have outlined below our responses to the comments by the reviewer and the academic editor.

**Responses to the academic editor**

Journal Requirements:

When submitting your revision, we need you to address these additional requirements.

1. Please ensure that your manuscript meets PLOS ONE's style requirements, including those for file naming.

Response: Thank you for this comment. We have reviewed the style requirements and confirm that the manuscript should be in line.

2. Note from Emily Chenette, Editor in Chief of PLOS ONE, and Iain Hrynaszkiewicz, Director of Open Research Solutions at PLOS: Did you know that depositing data in a repository is associated with up to a 25% citation advantage (https://doi.org/10.1371/journal.pone.0230416)? If you’ve not already done so, consider depositing your raw data in a repository to ensure your work is read, appreciated and cited by the largest possible audience. You’ll also earn an Accessible Data icon on your published paper if you deposit your data in any participating repository (https://plos.org/open-science/open-data/#accessible-data).

3. In the online submission form, you indicated that [Data cannot be shared publicly because of containing pseudonymised personal data. Data are available upon reasonable request for researchers who meet the criteria for access to confidential data by contacting the corresponding author. The syntaxes used for data analysis will be available on

DANS: https://doi.org/10.17026/SS/NIDSIJ].

All PLOS journals now require all data underlying the findings described in their manuscript to be freely available to other researchers, either 1. In a public repository, 2. Within the manuscript itself, or 3. Uploaded as supplementary information.

This policy applies to all data except where public deposition would breach compliance with the protocol approved by your research ethics board. If your data cannot be made publicly available for ethical or legal reasons (e.g., public availability would compromise patient privacy), please explain your reasons on resubmission and your exemption request will be escalated for approval.

Response: Thank you for pointing this out. We are aware of the benefits of open science and publicly available data, and fully agree with you. We have managed to anonymise our data in line with guidelines and restrictions of both Dutch legal and hospital (ethical) policies. The dataset has now been uploaded to DANS along with the syntaxes. Our data availability statement has been updated as follows:

“The study data and syntaxes are available in the DANS repository: <https://doi.org/10.17026/SS/NIDSIJ>. The dataset has been fully anonymised and demographic and clinical characteristics (used only for descriptive analysis of the sample) have been obfuscated, as they comprise sensitive data that could compromise patient privacy. The key to these characteristics is available upon reasonable request by contacting the corresponding author.”

4. Please review your reference list to ensure that it is complete and correct. If you have cited papers that have been retracted, please include the rationale for doing so in the manuscript text, or remove these references and replace them with relevant current references. Any changes to the reference list should be mentioned in the rebuttal letter that accompanies your revised manuscript. If you need to cite a retracted article, indicate the article’s retracted status in the References list and also include a citation and full reference for the retraction notice.

Response: The references have been checked to ensure that all citations are correct, refer to the most recent version, and have not been retracted. One citation has been altered to refer to a more recent version (*Lopez-Leon et al., 2021*). Additionally, three citations have been slightly revised to better fit the journal reference style requirements.

**Responses to the reviewer**

Reviewer #1: The authors have attempted to study one of the interesting concepts of positive and negative affect and its association with post COVID symptoms. Following suggestions shall be considered

We thank the reviewer for the thoughtful and constructive feedback, which helped us improve our manuscript.

1)As per author’s description, it seems that Post-COVID symptom diagnosis is patient perceived (no clarity on clinician’s involvement in the same), so it shall be explicitly mentioned as “Self-reported post COVID symptoms”.

Response: Post-COVID-19-syndrome was indeed self-assessed (as described under Recruitment), and refers to self-reported symptoms that were attributed by the participants to this syndrome and not attributable to health conditions prior to COVID-19 and hospitalisation. Throughout the manuscript, we have added ‘self-reported’ where this might have led to confusion, or have ensured that this should be clear to readers.

2)Did patients were oriented towards the questionnaire/ESM? It is important to mention in methodology section.

Response: We agree we you that this is important information. Participants were instructed and informed on the nature of ESM in this study, as well as the use of the application. We have added a paragraph explaining this in the Procedure section:

“*Before the data collection started, participants were carefully instructed on how to install and set up the application, on how the questionnaires worked, and on what was expected from them (i.e. to respond to as many questionnaires as possible). Participants could practice with the questionnaire before the study started. Finally, participants were informed to contact the researchers if and when necessary before the start of and during the data collection period*.”

3) Also, as post COVID symptom is a cluster term imbibing numerous conditions, a detailed information regarding type of post COVID symptom and clinical condition of the patients, type and duration of comorbidity, management of comorbidity needs to be provided in the result section.

Response: Thank you for this comment. We have not collected in-depth information on all (self-)reported signs and symptoms attributed to post-COVID-19 syndrome, as this was beyond the scope of our study. The symptoms of interest in our study (physical and mental fatigue, dyspnoea, cognitive dysfunction and pain, as well as negative affect) were based on prior research (Schaap et al., 2022) in which no other post-COVID-19-related symptoms were described as being present at that time by our participants. As described in our introduction, findings in meta-analyses supports the decision to focus on the selected symptoms.

Furthermore, participants were selected on self-assessment of post-COVID-19 syndrome, including self-attribution of the symptoms to long-term sequelae of COVID-19. As the influence of (some) comorbidities on post-COVID-19 syndrome is yet unclear, we have reported these. In our overarching project (see Clinical Trials NCT05813574), we will investigate the potential influence of comorbidities in COVID-19 survivors, but this falls beyond the scope of the current study. We have now added the comorbidities in the Results section:

“*Reported comorbidities were chronic respiratory condition (n = 2), gastrointestinal disease (n = 2), hypertension (n = 2), hypothyroidism (n = 1), cancer survivor (n = 1), cardiovascular (n = 1), diabetes mellites (n = 1), rheumatic disease (n = 1).“*
