# Supplementary material for: Affect and post-COVID-19 symptoms in daily life: An exploratory experience sampling study
Source: PLoS One. 2024 Oct 24;19(10):e0295217. doi: 10.1371/journal.pone.0295217 (PMC11500883; doi:10.1371/journal.pone.0295217)
Supplement: Attachment — Submitted filename: Responses to Reviewers.docx [file pone.0295217.s006.docx]

Thank you again for your time and for inviting us to revise our manuscript, *Affect and post-COVID-19 symptoms in daily life: An exploratory experience sampling study*. We have outlined below our responses to the comments of the reviewers in blue. As we have included citations in our responses, we have added a reference list at the end of this document.

**Responses to the reviewer #2**

We thank the reviewer for the critical feedback, which helped us in reflecting on the quality of our study and the interpretability of our methodological approaches.

Reviewer #2: Numerous publications have extensively covered this topic (many on PlosONE too). It is well-established that these symptoms exhibit correlations. However, it seems unlikely that this manuscript will contribute novel insights to the existing research.

Although there are indeed a good number of studies on post-COVID-19 syndrome, insights into patient daily life experiences in their illness as well as suitable treatment options beyond symptom management are still lacking. So far, there are (still) no published studies on the role of affect/emotions or emotional well-being on post-COVID-19 syndrome in general and post-COVID symptoms in specific, despite evidence of the relationship between affect and short-term symptom severity (as well as for long-term recovery and survival; see e.g. Lamers et al., 2012) suggesting the importance and potential of these observations. As discussed in the discussion, this information can be used for new treatments, such as developing or applying positive affect skills training or other positive psychological interventions, which might contribute to improvements in emotional well-being, short-term symptom relief and long-term self-management skills development.

The sample size is notably limited, rendering the results unreliable for any statistical model. It may be appropriate to perform descriptive analysis only. A general guideline recommends having one covariate per 10 subjects.

While we agree with the reviewer that the sample of participants is on the smaller side – and hence explorative in nature – we would like to point out that these general guidelines are specifically for simple or generalised linear models and not applicable for multilevel (or hierarchical) linear models, which include many observations that are not aggregated on a sample level. Consequently, we would argue that the sample *of observations* in the study is sufficient for the regression models. One study showed that 2 subjects (or events or observations) per independent variable in ‘simple' (non-multilevel/not repeated) linear regression models are sufficient (see Austin & Steyerberg, 2015; cf. van Smeden et al., 2016). In total, our dataset included 502 observations (see line 191, p. 8).

Moreover, as our focus is on exploring the relationships, we primarily approached our analysis in a qualitative way by interpreting the various graphs. To aid in the interpretation of our findings, we also provide the statistical models, which are in line with what we observe in the qualitative visual approach. Both approaches taken together make us feel conformable in the accuracy of our findings (although still explorative; these should not be generalised without care).

It's worth noting that the Spearman correlation does not account for within-subject correlation over time; it may not be appropriate to combine timepoints together.

Thank you for pointing this out to us! It was not our intention to imply that that was what we aimed at; instead, this part of the analysis focused on between-subject relationships, namely whether a general higher severity of (e.g.) physical fatigue was associated with a general higher severity of dyspnoea. Within-subject fluctuations were considered using a qualitative approaches (see Figure 1) We decided on this approach, as we thought that multilevel regression models provided a distorted picture; these correlation analyses are more conservative in the estimation (i.e. less impacted by variation) and suited our interests (namely: to what extent do symptoms co-occur) better.

We have rewritten the sentence to clarify why and how we have applied Spearman correlations.

*“Statistical evidence for associations between symptoms in general were explored using personal averages (Person Mean) of the severity of each symptom within each participant. Spearman rank correlations (r_s_) were used to account for non-parametric distributions and the small sample size.”* (p. 7, lines 155-158)

In Figure 1, the pain curves display limited variation, making it challenging to believe in significant correlations with other symptoms.

We agree with the reviewer that pain did indeed not show a lot of temporal fluctuation. Pain was usually either present or absent on a daily level within participants. Any variability is difficult to observe in the graph, as these are presented in steps of 0.17 per day (e.g. 1 for pain on all measurements of the day, 0.8 for pain on 5 out of 6 measurements of the day).

However, as the correlations suggest, this does not disagree with the finding that when participants tend to suffer from pain (i.e. a personal mean closer to 1), they also had a tendency to score higher on other symptom severity scores. As we have acknowledged in the Methods section (p. 7, lines 133-139), our measurement of pain was far from ideal, but we think that including a measure of pain presence is preferable over ignoring this commonly reported post-COVID symptom in our analysis.

**Responses to the reviewer #3**

Reviewer #3: The authors investigated the daily courses and interrelations between positive and negative affect and post-COVID-19 symptom severity by using an ESM approach. The authors demonstrated that post-COVID-19 symptoms were found to fluctuate in severity both within and between days, and these severities fluctuated in co-occurrence with each other. The topic is important and interesting. This is a revised version and the authors have taken the suggestions into consideration and the manuscript has improved. The clinical condition of the patients can be a factor impact the post COVID19 symptom and the authors listed comorbidities in the Results section. No major concerns were detected in the manuscript. It would be great if the authors can discuss why MLR models were selected for the analysis.

We thank the reviewer for the kind compliments and the great question regarding the choice of analysis. We have revisited the data analysis section and added some references to handbooks that address how ESM data can and should be addressed for the sake of conciseness (p. 7, lines 161-163). We also would like to provide a more detailed answer to your question below.

MLR are extensions of (general) linear models that can account for multiple levels of data, that is, for example, data that is nested within a higher level of data. In this case, observations are nested within individuals. These models can also be referred to as hierarchical linear models. For ESM data, this means that they allow for modelling data with repeated measures – and hence not aggregating all measurements within one participant or on a sample level – and handling missing data through a Maximum Likelihood estimation (i.e. estimating the highest possibility of the likely value of a certain missing observation). Moreover, these models are flexible in that they allow for, e.g., modelling time or including lagged variables in autoregressive models, like we have done with our lagged association analyses. Using MLR models is a common analytical approach of analysis in ESM (Conner & Lehman, 2012; Myin-Germeys & Kuppens, 2022) and has likewise been used in similar ESM studies, such as by Brys et al. (2020) and Lenaert et al. (2020), as well as in our companion study (Wensink et al., 2023). For specific information on how MLR can be used with SPSS, their website (<https://www.ibm.com/docs/en/spss-statistics/29.0.0?topic=statistics-linear-mixed-models>) provides a clear overview.

**References**

Austin, P. C., & Steyerberg, E. W. (2015). The number of subjects per variable required in linear regression analyses. *Journal of Clinical Epidemiology*, *68*(6), 627-636. <https://doi.org/https://doi.org/10.1016/j.jclinepi.2014.12.014>

Brys, A. D. H., Stifft, F., Van Heugten, C. M., Bossola, M., Gambaro, G., & Lenaert, B. (2020). Unraveling Fatigue in Hemodialysis Patients: Comparing Retrospective Reports to Real-Time Assessments With an mHealth Experienced Sampling Method. *Journal of Pain and Symptom Management*, *60*(6), 1100-1108.e1102. <https://doi.org/10.1016/j.jpainsymman.2020.06.042>

Conner, T. S., & Lehman, B. J. (2012). Launching a Study in Daily Life. In M. Mehl & T. S. Conner (Eds.), *Handbook of Research Methods for Studying Daily Life* (pp. 88 - 107). Guilford Press.

Lamers, S. M. A., Bolier, L., Westerhof, G. J., Smit, F., & Bohlmeijer, E. T. (2012). The impact of emotional well-being on long-term recovery and survival in physical illness: a meta-analysis. *Journal of Behavioral Medicine*, *35*(5), 538-547. <https://doi.org/10.1007/s10865-011-9379-8>

Lenaert, B., Neijmeijer, M., van Kampen, N., van Heugten, C., & Ponds, R. (2020). Poststroke Fatigue and Daily Activity Patterns During Outpatient Rehabilitation: An Experience Sampling Method Study. *Archives of Physical Medicine and Rehabilitation*, *101*(6), 1001-1008. <https://doi.org/10.1016/j.apmr.2019.12.014>

Myin-Germeys, I., & Kuppens, P. (Eds.). (2022). *The open handbook of experience sampling methodology: A step-by-step guide to designing, conducting, and analyzing ESM studies* (2nd ed.). Center for Research on Experience Sampling and Ambulatory Methods Leuven. <https://www.kuleuven.be/samenwerking/real/real-book>.

van Smeden, M., de Groot, J. A. H., Moons, K. G. M., Collins, G. S., Altman, D. G., Eijkemans, M. J. C., & Reitsma, J. B. (2016). No rationale for 1 variable per 10 events criterion for binary logistic regression analysis. *BMC Medical Research Methodology*, *16*(1), 163. <https://doi.org/10.1186/s12874-016-0267-3>

Wensink, M., Schaap, G., ten Klooster, P. M., Doggen, C. J. M., van der Palen, J., Vonkeman, H. E., & Bode, C. (2023). Physical and mental fatigue in post-COVID syndrome and their associations over time: A small-sample ESM-study to explore fatigue, quality of sleep and behaviours. *Journal of Psychosomatic Research*, *164*, 111084. <https://doi.org/10.1016/j.jpsychores.2022.111084>
