# Supplementary material for: Affect and post-COVID-19 symptoms in daily life: An exploratory experience sampling study
Source: PLoS One. 2024 Oct 24;19(10):e0295217. doi: 10.1371/journal.pone.0295217 (PMC11500883; doi:10.1371/journal.pone.0295217)
Supplement: Attachment — Submitted filename: Response to reviewers.docx [file pone.0295217.s007.docx]

Dear editors,

Herewith, we submit a formal appeal to the rejection of our manuscript, titled Affect and post-COVID-19 symptoms in daily life: An exploratory experience sampling study.

In our opinion, the rejection is based on several mistakes. After submitting the manuscript on 15 November 2023, we were originally invited by the editor (on 4 January) to address some minor revisions to the manuscript and to make our dataset publicly available. After complying accordingly with the remarks of the editor and reviewer, the editor became unavailable. On 3 May, we received another round of feedback from reviewers, which was surprising to us, as we thought the manuscript was all but accepted. Moreover, based on the comments we received, we do not think that these reviewers were able to correctly assess the study on its merits.

Below we respond to the reasons provided (marked in blue) point by point:

1. The study's results may not be generalizable due to the small sample size, with only 10 participants included in the analysis.

We agree with the editor that the sample size is small, and we have acknowledged this in our cover letter. Our study is explorative in nature (indicated in the title) as experience sampling methodology (ESM) studies often are. Our aim was to provide some insight into the daily life of people with post-COVID-19 syndrome, and generalisability to wider populations was not our objective. We argue that this is the start of future research in a field that requires more attention (namely the relationship between (psycho)somatic symptoms and affect/emotional well-being in daily life). Moreover, we have indicated the small sample size as a limitation in our discussion.

2. Previous publications with larger datasets have already established high correlations among the symptoms, making this study not particularly novel.

We also acknowledge this statement. However, this correlational analysis was not an objective of our study, and served to provide context for the main analyses: the fluctuation of post-COVID symptoms in daily life and the association between these symptoms and affect over time.

3. The study lacks baseline data for the sample and is primarily descriptive in nature.

Baseline characteristics have been provided in our manuscript (page 8), but were other than descriptive not of interest for our objectives, that is, not relevant for our qualitative or statistical assessments. This criticism has not been provided to us by reviewers before.

As mentioned in point 1, we acknowledge that our study is mostly descriptive. To our awareness, PLOS ONE does not restrict accepting descriptive studies, as other ESM studies but also, for example, qualitative studies have been published here before.

4. The study does not meet the guideline of at least 10 subjects per factor.

As we have outlined in our responses to the reviewers, this guideline is not only arbitrary and strongly debated for linear models, it does not apply to multilevel models such as multilevel linear regression, which require repeated observations. Moreover, the editor has not acknowledged that our analysis is primarily qualitative, and the statistical models support our interpretation of the data. This argument was also provided in our response to the reviewers.

5. The methodology used for the small sample size is questionable.

There are no guidelines for sample sizes in ESM studies, as the number of sampled observations can vary wildly. Key is that the number of observations should be sufficient for the purpose (although here too there are no guidelines for what that entails). With 750 included observations, we think this holds true for an explorative study. We do not test hypotheses, but have set out to explore (co)variation of symptoms and affect qualitatively and statistically.

6. It is advised that the authors address these concerns by collecting more data and analyzing it according to the recommendations provided by the reviewers.

As has been outlined above, we respectfully disagree with the recommendations provided by the reviewers and editor.

One of the critiques from the reviewer that the author addressed is that the study is beyond its scope. The author mentioned that "in-depth information on all (self-)reported signs and symptoms attributed to post-COVID-19 syndrome is beyond the scope of our study." This statement should be included as a limitation in the manuscript. However, while reading the second revision, this statement was not included. They also need to include a table with the baseline characteristics of the sample and provide a flow diagram of the subjects.

We agree with the editor that we should have acknowledged this as a potential limitation in our discussion. We have added this in the limitation section (p. 15 of the attached revised manuscript). The requirements to include baseline characteristics as a table (and not an in-text description as now is the case) and a flow diagram have not been mentioned before. We could therefore not have included these in our revisions. For the sake of conciseness, we have not added these to the revised manuscript, but are willing to comply if the editor and reviewers think this would improve the manuscript.

Best regards,

Gerko Schaap, Marleen Wensink, Carine Doggen, Job van der Palen, Harald Vonkeman, and Christina Bode
